# Retroperitoneal Bronchogenic Cyst: A Rare Diagnosis of a Retroperitoneal Mass

**DOI:** 10.7759/cureus.76922

**Published:** 2025-01-04

**Authors:** Maryum Qureshi, Stefan Wawryk, Zeng Yap

**Affiliations:** 1 General Surgery, Austin Health, Melbourne, AUS; 2 Pathology, Dorevitch Pathology, Melbourne, AUS; 3 Endocrine and General Surgery, Werribee Mercy Hospital, Melbourne, AUS

**Keywords:** bronchogenic cyst, embryogensis, foregut anomaly, rare, retroperitoneal mass

## Abstract

Bronchogenic cysts (BCs) are rare clinical entities caused by the abnormal budding of the tracheobronchial tree during embryogenesis. This foregut anomaly is usually found in the thorax. Retroperitoneal bronchogenic cysts (RBCs) are extremely uncommon and usually located near the left adrenal gland. A definitive diagnosis can only be reached with surgical resection and histopathology. We report the case of a 52-year-old female with an RBC found incidentally on CT imaging and managed with surgical resection. Despite the rarity of RBCs, they should be considered in the differential diagnosis of patients with a retroperitoneal mass. Surgery is the standard treatment of choice. This case report is unique as it describes the rare occurrence of an RBC located adjacent to the right kidney.

## Introduction

Bronchogenic cysts (BCs) are congenital anomalies arising from abnormal budding of the tracheobronchial tree during the third to seventh week of embryonic development [1,2]. They are usually benign masses located in the mediastinum or lung parenchyma. BCs are frequently asymptomatic but can present with symptoms due to mass effects or secondary complications such as infection, haemorrhage, perforation, and, in rare cases, malignancy [2]. Useful imaging modalities include CT and MRI, with the lesion appearing as a well-defined mass adjacent to or involving the adrenal glands [1,2,3]. The absence of pathognomonic imaging features in BCs makes their preoperative diagnosis challenging. BCs in the retroperitoneum are very rare with only 65 cases reported in the literature in English so far. We report the case of a 52-year-old female with an incidental finding of a right-sided retroperitoneal bronchogenic cyst (RBC) confirmed on histology post-surgical resection.

This report was previously presented as a poster at the Royal Australasian College of Surgeons 92nd Annual Scientific Congress on May 6-10th, 2024.

## Case presentation

**Figure 1:**
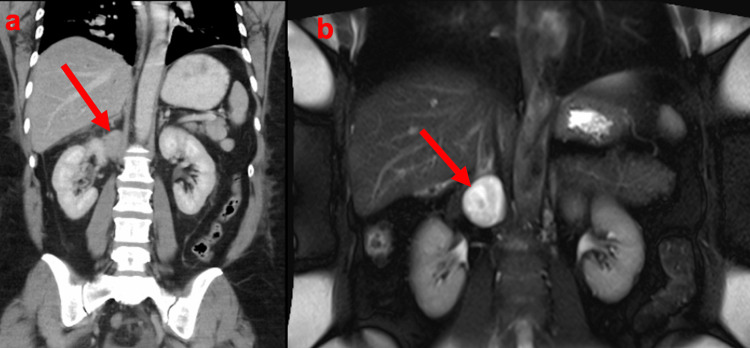
(a) Coronal CT and (b) MRI showing a 28 mm soft tissue mass (RBC – red arrow) adjacent to the right kidney CT: computed tomography; MRI: magnetic resonance imaging; RBC: retroperitoneal bronchogenic cyst

The patient was a 52-year-old female with an incidental finding of a right retroperitoneal mass discovered during follow-up imaging after choledochal cyst surgery. The choledochal cyst was diagnosed via intraoperative cholangiogram performed during a laparoscopic cholecystectomy for biliary colic. The patient had no other significant comorbidities and no other surgical history. Following the choledochal cyst surgery, subsequent CT scans revealed a 28 mm soft tissue mass of uncertain aetiology (Figure 1a).

**Figure 2:**
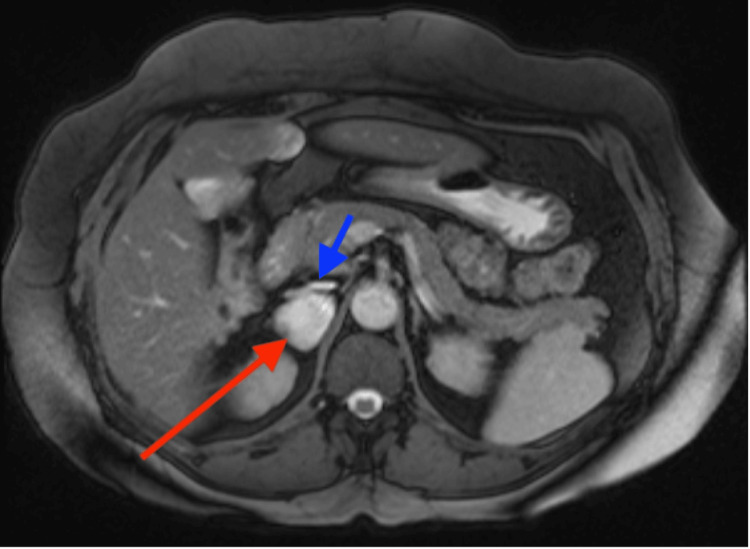
Axial T2 MRI demonstrating the IVC (blue arrow) being compressed by the RBC (red arrow) IVC: inferior vena cava; MRI: magnetic resonance imaging; RBC: retroperitoneal bronchogenic cyst

The mass was located above the right kidney and posterior to the inferior vena cava. Initial differential diagnoses included retroperitoneal lymphadenopathy or an adrenal mass. An MRI was performed to further characterise the lesion. The mass was hyperintense on both T1- and T2-weighted sequences with no evidence of diffusion restriction (Figures 1b, 2). Possible differentials included lymphangioma, leiomyoma, and a retroperitoneal cyst. Given the location and imaging characteristics, there was concern for a possible adrenal lesion. An endocrine workup including plasma metanephrines to rule out phaeochromocytoma, aldosterone: renin ratio to exclude primary hyperaldosteronism, and a low-dose overnight dexamethasone suppression test to evaluate for Cushing’s syndrome were performed. All these tests were negative.

The patient underwent serial imaging over a period of two years to monitor the lesion. During this time, the mass increased in size from 29 x 20 mm to 27 x 35 mm. Given the growth of the lesion and the diagnostic uncertainty, a decision for surgical intervention was made. A retroperitoneoscopic resection of the mass was performed. Intraoperatively, the lesion was separate from the right adrenal gland and appeared cystic in nature. The mass was successfully removed in its entirety; the patient recovered well without complication and was discharged two days later.

**Figure 3:**
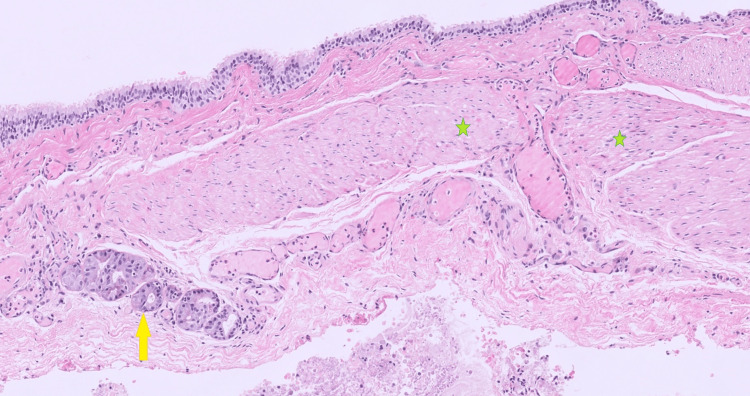
Histological findings The image shows a unilocular cyst lined by ciliated pseudostratified columnar cells with discontinuous fascicles of smooth muscle (green stars) and scattered seromucinous glands (yellow arrow); H&E, x10 magnification

Histopathology revealed a 30 x 25 x 7 mm cyst lined by pseudostratified ciliated columnar epithelium, with fascicles of smooth muscle in the cyst wall associated with scattered seromucinous glands. Cartilage was not seen; no malignant cells were identified. The histological findings were consistent with an RBC (Figure 3).

## Discussion

BCs are rare clinical entities caused by abnormal budding of the tracheobronchial tree during early embryonic development. The tracheobronchial tree originates from the foregut [4,5]. The fusion of the pleuroperitoneal membrane divides the pericardial-peritoneal canal into two cavities, leading to the formation of the diaphragm. During this process around the sixth week of embryological development, bronchial trees can be pinched off these membranes leading to ectopic locations of bronchial-derived tissue which can develop into BCs as the embryo matures [4,5].

Typically, these lesions are found in the thorax. In rare cases, they can occur in ectopic locations with RBCs being extremely uncommon [1,2]. Our case is noteworthy due to the rare occurrence of an RBC located adjacent to the right kidney; most reported RBCs are found near the left adrenal gland [2]. RBCs were first reported by Miller et al. in 1953 [2]. An extensive PubMed search revealed 65 case reports of RBCs documented in the literature in English to date. They have been reported to occur almost equally in men and women [1].

The clinical presentation of RBCs is variable. Most are asymptomatic and often found incidentally during imaging for other reasons, as in this case. Symptoms usually occur due to mass effects on adjacent structures or complications such as infection, bleeding, or perforation [1-3]. Symptoms include abdominal or back pain, nausea, vomiting, and, rarely, hypertension due to compression of the adrenal gland [6]. There is a broad range of differential diagnoses for RBCs, including cystic lymphangioma, cystic mesothelioma, cystic teratoma, epidermoid cyst, tailgut cyst, bronchopulmonary sequestration, and cysts of urothelial and Mullerian origin [1]. They can be easily misdiagnosed as adrenal tumours, cysts, or adrenal TB when located near the adrenal gland, as in the above case [7]. This emphasises the importance of a thorough diagnostic workup, including appropriate endocrine testing and advanced imaging.

Imaging plays a key role in RBC diagnosis. On CT, RBCs can appear as homogenous, hypoattenuating lesions lacking enhancement following contrast administration, with possible calcification of the cyst wall. However, CT appearance can be variable due to cyst content. High mucoid or proteinaceous content in the cyst can appear hyperattenuating, mimicking a solid mass. MRI can help characterise the true cystic nature of the lesion. BCs usually show a high to medium-intensity signal on T1-weighted images and a high-intensity bright signal on T2-weighted images [1,8]. In lesions located close to the pancreas, endoscopic ultrasound (EUS) can identify characteristic features of RBCs such as their hypoechoic nature, and fine-needle aspiration can be employed to obtain cyst fluid for cytological analysis [6]. Despite their characteristic imaging features, these lesions can still be misdiagnosed. A definitive diagnosis requires histopathology of the lesion. Hallmark features of BCs include well-defined bronchial cysts lined with pseudostratified ciliated columnar epithelium with bronchial glands, smooth muscle, cartilage, and mucoid material [9,10].

Surgical excision remains the gold standard for both diagnosis and treatment of BCs [3,9]. The decision for operative management is based on several factors, including the presence of symptoms, rate of growth over time, and uncertain benignity of the lesion. While the rate of growth of RBCs is not well described due to its rarity, resection of enlarging cysts aims to alleviate symptoms if present and prevent complications such as infection, haemorrhage, rupture, and, rarely, malignant transformation [2,3].

Malignancy in RBCs is rare with an estimated rate of less than 1% [11]. However, as imaging appearances of RBCs are non-specific and can mimic benign conditions such as cystic teratomas or adrenal masses, surgical excision is required for histopathological diagnosis [11]. Recently, minimally invasive options such as laparoscopic transperitoneal and retroperitoneoscopic techniques have been utilised with excellent outcomes [12]. The retroperitoneoscopic approach is superior to the laparoscopic transperitoneal approach due to reduced operative time, less pain, and shorter hospital stays [13]. If the cyst is adherent to surrounding structures, it is recommended to cauterise the residual cyst wall to prevent recurrence [1,3,12,14].

## Conclusions

Although RBCs are rare, they should be considered in the differential diagnosis of a retroperitoneal mass, particularly if located near the adrenals or adjacent structures. Even with imaging, preoperative diagnosis can be challenging and requires a multi-disciplinary approach. Surgery is the recommended treatment of choice as only histopathology can provide a definite diagnosis.
